# Filamentous invasive growth of mutants of the genes encoding ammonia-metabolizing enzymes in the fission yeast *Schizosaccharomyces pombe*

**DOI:** 10.1371/journal.pone.0186028

**Published:** 2017-10-05

**Authors:** Yoshie Sasaki, Ayumi Kojima, Yuriko Shibata, Hiroshi Mitsuzawa

**Affiliations:** 1 Department of Applied Biological Science, College of Bioresource Sciences, Nihon University, Fujisawa, Kanagawa, Japan; 2 Department of Food Bioscience and Biotechnology, College of Bioresource Sciences, Nihon University, Fujisawa, Kanagawa, Japan; 3 Department of Bioscience in Daily Life, College of Bioresource Sciences, Nihon University, Fujisawa, Kanagawa, Japan; University of Cambridge, UNITED KINGDOM

## Abstract

The fission yeast *Schizosaccharomyces pombe* undergoes a switch from yeast to filamentous invasive growth in response to certain environmental stimuli. Among them is ammonium limitation. Amt1, one of the three ammonium transporters in this yeast, is required for the ammonium limitation-induced morphological transition; however, the underlying molecular mechanism remains to be understood. Cells lacking Amt1 became capable of invasive growth upon increasing concentrations of ammonium in the medium, suggesting that the ammonium taken up into the cell or a metabolic intermediate in ammonium assimilation might serve as a signal for the ammonium limitation-induced morphological transition. To investigate the possible role of ammonium-metabolizing enzymes in the signaling process, deletion mutants were constructed for the *gdh1*, *gdh2*, *gln1*, and *glt1* genes, which were demonstrated by enzyme assays to encode NADP-specific glutamate dehydrogenase, NAD-specific glutamate dehydrogenase, glutamine synthetase, and glutamate synthase, respectively. Growth tests on various nitrogen sources revealed that a *gln1*Δ mutant was a glutamine auxotroph and that a *gdh1*Δ mutant had a defect in growth on ammonium, particularly at high concentrations. The latter observation indicates that the NADP-specific glutamate dehydrogenase of *S*. *pombe* plays a major role in ammonium assimilation under high ammonium concentrations. Invasive growth assays showed that *gdh1*Δ and *glt1*Δ mutants underwent invasive growth to a lesser extent than did wild-type strains. Increasing the ammonium concentration in the medium suppressed the invasive growth defect of the *glt1*Δ mutant, but not the *gdh1*Δ mutant. These results suggest that the nitrogen status of the cell is important in the induction of filamentous invasive growth in *S*. *pombe*.

## Introduction

Many fungi undergo a morphological transition from yeast to filamentous growth in response to changes in environmental conditions, such as ammonium limitation. It has been reported for a variety of fungi [1–5] that the ammonium limitation-induced morphological transition requires certain members of the Amt/Mep/Rh family of ammonium transport proteins [6].

The budding yeast *Saccharomyces cerevisiae* has three ammonium transporters, Mep1, Mep2, and Mep3 [7], and the high-affinity transporter Mep2 is required for diploid pseudohyphal growth in response to ammonium limitation [1]. It has been proposed that Mep2 acts as an ammonium sensor that generates a signal for the morphological transition [1]. Two signal transduction pathways, the MAP kinase and cAMP–PKA pathways, seem to be involved in the regulation of the pseudohyphal growth [8, 9]. The mechanism by which Mep2 senses ammonium and activates the downstream pathways, however, remains to be elucidated.

The fission yeast *Schizosaccharomyces pombe* also has three ammonium transporters, Amt1, Amt2, and Amt3, which possess different uptake capacities [4]. The presumed high-affinity transporter Amt1 is required for ammonium limitation-induced filamentous invasive growth. Two models have been suggested for the role of Amt1 in the induction of the filamentous invasive growth in *S*. *pombe*. Amt1 may function as a sensor, as has been proposed for *S*. *cerevisiae* Mep2. Another possibility is that ammonium taken up into the cell may act to induce the morphological transition [4]. Lowering the ammonium concentration in the medium, like deleting the *amt1* gene, leads to reduced surface growth and a defect in invasive growth, suggesting that invasive growth requires some level of intracellular ammonium and that the invasive growth defect of cells lacking Amt1 is ascribed to a defect in high-affinity ammonium uptake. Thus, the intracellular level of ammonium or a metabolite produced through ammonium assimilation might play an important role in the ammonium limitation-induced morphological transition of *S*. *pombe*.

Two pathways operate for ammonium assimilation in *S*. *cerevisiae* [10, 11]. NADP-specific glutamate dehydrogenase (NADP-GDH) converts ammonium and 2-oxoglutarate to glutamate. Glutamine synthetase (GS) forms glutamine from ammonium and glutamate with the consumption of ATP, which is followed by synthesis of glutamate from glutamine and 2-oxoglutarate by glutamine oxoglutarate aminotransferase (GOGAT) (also called glutamate synthase). NAD-specific glutamate dehydrogenase (NAD-GDH) has a catabolic role. It catalyzes the formation of ammonium and 2-oxoglutarate from glutamate, the reverse reaction of that catalyzed by NADP-GDH.

In this study, we show that *S*. *pombe* Amt1 becomes dispensable for the filamentous invasive growth if the concentration of ammonium in the medium is increased. As an attempt towards elucidating a possible role of intracellular ammonium or a metabolic intermediate as a signal to induce the induction of the filamentous invasive growth, deletion mutants were constructed for genes encoding ammonia-metabolizing enzymes. Some of the mutants exhibited a defect in the morphological transition.

## Materials and methods

### Strains

The *S*. *pombe* strains used in this study are listed in Table 1. FY7651 (*h*^-^ *ura4-D18*) and FY7406 (*h*^90^ *ura4-D18*) were the parent strains for gene disruption. FY7507 was used as a source of *ura4*^+^ as described previously [4]. A 2.9-kb *ura4*^+^ fragment was amplified by PCR to replace the *ura4-D18* allele of FY7651, yielding the prototrophic strain HMP126.

**Table 1:** *S*. *pombe* strains used in this study.

Strain	Genotype	Source or reference
*h*^-^ strains		
FY7651 (MM72-13B)	*h*^-^ *ura4-D18*	NBRP^a^ (Yeast)
FY7507 (L972)	*h*^-^	Mitsuzawa (2006)
HMP126	*h*^-^	This study
HMP135	*h*^-^ *ura4-D18 gdh1*Δ::*ura4*^+^	This study
HMP128	*h*^-^ *ura4-D18 gdh2*Δ::*ura4*^+^	This study
HMP141	*h*^-^ *ura4-D18 gln1*Δ::*ura4*^+^	This study
HMP125	*h*^-^ *ura4-D18 glt1*Δ::*ura4*^+^	This study
*h*^90^ strains		
FY7406	*h*^90^ *ura4-D18*	Mitsuzawa (2006)
HMP94	*h*^90^	Mitsuzawa (2006)
HMP95	*h*^90^ *amt1*Δ	Mitsuzawa (2006)
HMP137	*h*^90^ *ura4-D18 gdh1*Δ::*ura4*^+^	This study
HMP133	*h*^90^ *ura4-D18 gdh2*Δ::*ura4*^+^	This study
HMP143	*h*^90^ *ura4-D18 gln1*Δ::*ura4*^+^	This study
HMP131	*h*^90^ *ura4-D18 glt1*Δ::*ura4*^+^	This study

^a^National BioResource Project of the Ministry of Education, Culture, Sports, Science and Technology of Japan.

### Media

YE, EMM [12], and EMM containing 0.1, 1, 10, or 100 mM NH_4_Cl, 10 mM glutamate, or 10 mM glutamine, instead of 93 mM (5 g/L) NH_4_Cl, were used. LNB, a low-ammonium medium containing 0.38 mM (0.05 g/L) (NH_4_)_2_SO_4_, was prepared as described previously [13]. When necessary, (NH_4_)_2_SO_4_ in LNB was replaced by 5 or 10 mM NH_4_Cl. Solid media contained 2% agar.

### Construction of deletion mutants of the *gdh1*, *gdh2*, *gln1*, and *glt1* genes

Plasmids for disrupting the *gdh1*, *gdh2*, *gln1*, and *glt1* genes were constructed by ligating four fragments simultaneously with an In-Fusion cloning kit (Clontech). 5’-Upstream and 3’-downstream sequences, a *ura4*^+^ marker cassette [14], and the pUC19 vector were amplified by PCR, gel-purified, and assembled together to generate plasmids containing the *ura4*^+^ marker flanked by the upstream and downstream sequences of the genes to be disrupted. Using these plasmids as templates, fragments for replacement of the wild-type allele were PCR-amplified and used to transform FY7651 and FY7406 to uracil prototrophy, yielding *gdh1*Δ::*ura4*^+^, *gdh2*Δ::*ura4*^+^, *gln1*Δ::*ura4*^+^, and *glt1*Δ::*ura4*^+^ mutants (hereafter referred to as *gdh1*Δ, *gdh2*Δ, *gln1*Δ, and *glt1*Δ mutants, respectively). The *gln1*Δ and *glt1*Δ alleles lack the entire ORFs, and the *gdh1*Δ and *gdh2*Δ alleles contain only the last two and eight amino acids of the ORFs, respectively (S1 Fig). The correct disruption was doubly verified by PCR with external and internal primer pairs, which were used to confirm the presence of the disrupted allele and the absence of the wild-type allele, respectively (S2 Fig). Primers used in this study are listed in S1 Table.

### Preparation of crude extract

Cells were grown at 30°C to an OD_600_ of 0.5 in 250 ml of glucose minimal media containing 10 mM ammonium, 10 mM glutamate, or 10 mM glutamine as the nitrogen source, harvested, washed with H_2_O, and, if necessary, stored at −80°C until use. The cells (ca. 200 mg) were resuspended in 400 μl of extraction buffer, consisting of 100 mM potassium phosphate buffer (pH 7.6), 1 mM EDTA, 1 mM dithiothreitol, and protease inhibitors, and then disrupted with 1.3 ml of glass beads (0.5 mm in diameter) six times for 1 min using the Micro Smash cell disruptor (TOMY, Tokyo, Japan). Cell extracts were recovered through a small hole punched at the bottom of the tube, and the supernatant after centrifugation at 20,000 × *g* for 20 min was used as a crude extract for enzyme assay. Total protein concentration (typically ca. 20 mg/ml) was determined by the bicinchoninic acid method.

### Assay of enzyme activities

NADP-GDH and NAD-GDH activities were determined by the method of Doherty [15] with minor modifications. The NADP-GDH reaction mixture (1 ml) contained 78 mM potassium phosphate buffer (pH 7.6), 10 mM 2-oxoglutaric acid, 200 mM NH_4_Cl, 0.25 mM NADPH, and 20 μl of crude extract. The NAD-GDH reaction mixture is the same as the NADP-GDH reaction mixture except that it contained 78 mM potassium phosphate buffer (pH 7.0) and 0.25 mM NADH. 2-Oxoglutarate was omitted in the blanks. The reaction mixture was equilibrated at 40°C for 5 min before adding the crude extract to initiate the reaction. The absorbance at 340 nm (A_340_) was recorded every second for 90 seconds using a kinetics program for the Shimadzu UVmini-1240 spectrophotometer, and the decrease in A_340_ over the first 10 seconds, when the reaction proceeded linearly (S3 Fig), was used to calculate the enzyme activity.

GS activity was determined by the transferase assay of Shapiro and Stadtman [16] with minor modifications. The reaction mixture (400 μl) contained 100 mM MES-NaOH (pH 6.3), 20 mM hydroxylamine-HCl, 1 mM MnCl_2_, 20 mM potassium arsenate, 0.5 mM disodium ADP, 100 mM L-glutamine, and 20 μl of crude extract. Arsenate and ADP were omitted in the blanks. The reaction was initiated by the addition of crude extract, allowed to proceed for 30 min at 30°C, and stopped by the addition of 800 μl of a solution of 5.5% FeCl_3_∙6H_2_O and 2% trichloroacetic acid in 0.25 N HCl. Insoluble material was removed by centrifugation, and A_540_ was measured. The amount of γ-glutamyl hydroxamate produced was determined from a standard curve.

GOGAT activity was determined by the method of Roon *et al*. [17] with minor modifications. The reaction mixture (1 ml) contained 83 mM potassium phosphate buffer (pH 7.8), 0.2 mM NADH, 5 mM L-glutamine, 5 mM 2-oxoglutaric acid, and 20 μl of crude extract. Glutamine was omitted in the blanks. The reaction mixture was equilibrated at 40°C for 5 min before adding the crude extract to initiate the reaction. A_340_ was recorded every second for 90 seconds, and the decrease in A_340_ over the first 30 seconds, when the reaction proceeded linearly (S3 Fig), was used to calculate the enzyme activity.

### Invasive growth assay

Growth underneath the surface of the agar medium was tested as described previously [4].

## Results

### Effect of increased concentrations of ammonium on the invasive growth defect of an *amt1*Δ mutant

When grown on LNB agar medium, which contains 0.76 mM ammonium as the nitrogen source, *S*. *pombe* forms microcolonies of filamentously growing cells underneath the surface of the medium ([4] and S4 Fig), and the presumed high-affinity transporter Amt1 is required for this ammonium limitation-induced filamentous invasive growth [4]. It was also shown that the wild-type strain displays little invasive growth when the ammonium concentration in the medium is lowered from 0.76 to 0.1 mM [4]. These observations prompted us to test if increasing the ammonium concentration in the medium had an effect on the invasive growth defect of an *amt1* deletion (*amt1*Δ) mutant.

**Fig 1:**
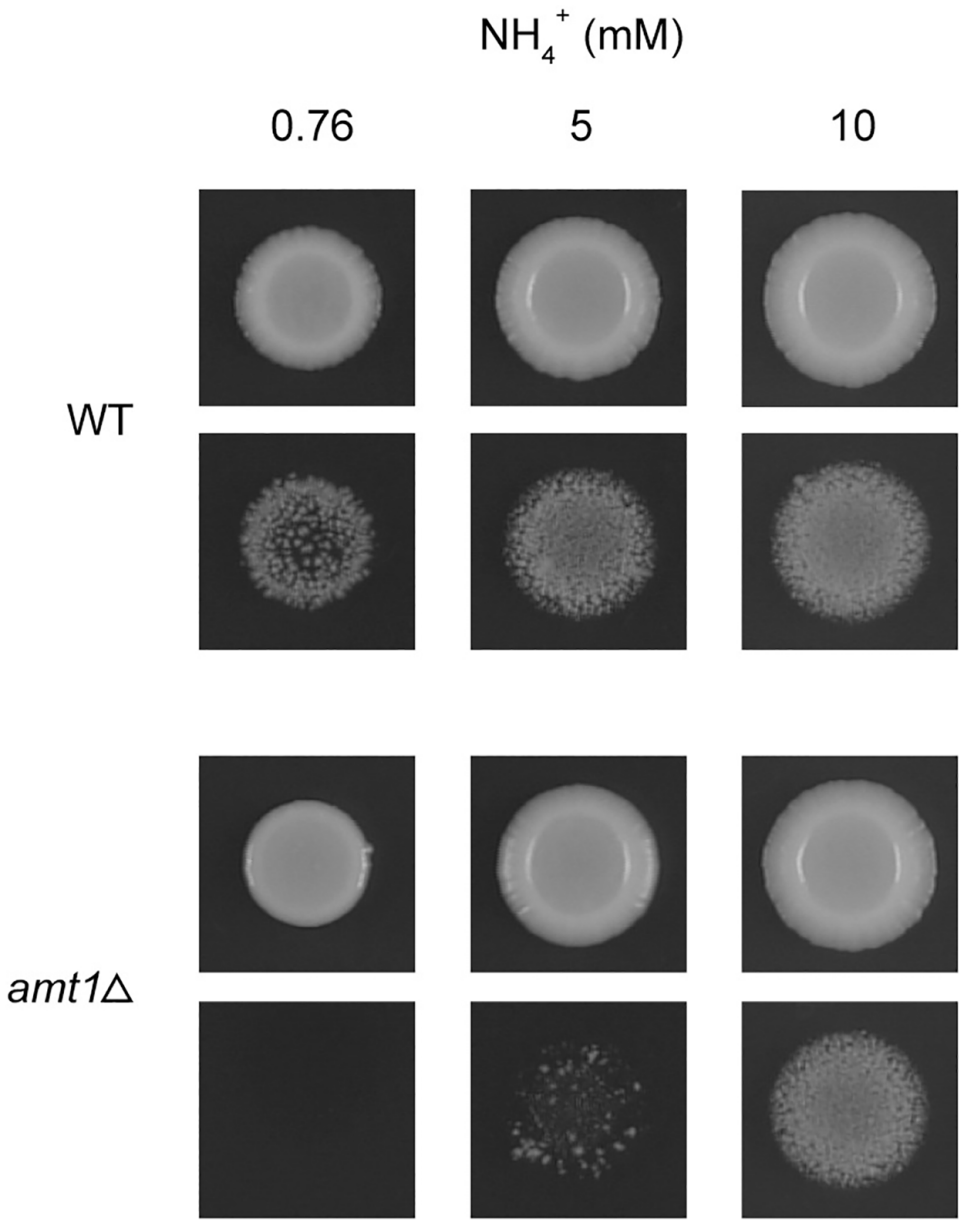
Invasive growth defect of an *amt1*Δ mutant and effect of increasing concentrations of ammonium. Five-microliter aliquots of cell suspension (5 x 10^4^ cells) were spotted onto LNB medium and media in which 0.76 mM NH_4_^+^ in LNB was replaced by 5 or 10 mM NH_4_^+^. The plates were incubated at 30°C for 14 days and photographed before (upper panels) and after (lower panels) the cells were washed off the surface of the agar. Strains are HMP94 (WT) and HMP95 (*amt1*Δ). All images are shown at the same magnification, so that size of the cell patches reflects growth rate. It is evident that increasing concentrations of ammonium enhance not only invasive growth but also surface growth.

As in the previous study, whereas a wild-type strain underwent invasive growth on a medium containing 0.76 mM ammonium, an *amt1*Δ mutant showed no invasive growth (Fig 1). When ammonium was present at 5 mM, the *amt1*Δ mutant exhibited a little invasive growth, and a higher concentration of ammonium (10 mM) resulted in more pronounced invasive growth (Fig 1). An enhancement of invasive growth by increasing concentrations of ammonium was also observed for the wild-type strain. At 10 mM ammonium, the *amt1*Δ mutant showed invasive growth comparable to that of the wild-type strain. These results indicate that Amt1 is dispensable for invasive growth at higher ammonium concentrations. While we cannot exclude the possibility that Amt2 or Amt3, through which ammonium is supposed to be taken up by the cell lacking Amt1, has a signaling function, the results described above are consistent with the hypothesis that intracellular ammonium or a metabolic intermediate in ammonium assimilation is a signal for inducing invasive growth.

### Construction of deletion mutants of genes involved in ammonia metabolism

As a first step toward identifying a signal for the invasive growth of *S*. *pombe*, we constructed deletion mutants of four genes, *gdh1* (SPCC622.12c), *gdh2* (SPCC132.04c), *gln1* (SPAC23H4.06), and *glt1* (SPAPB1E7.07), which are predicted to encode NADP-GDH, NAD-GDH, GS, and GOGAT, respectively [18]. The predicted *S*. *pombe* proteins show significant sequence similarity to the *S*. *cerevisiae* enzymes (S2 Table). As described in Materials and methods, *gdh1*Δ, *gdh2*Δ, *gln1*Δ, and *glt1*Δ mutants that lack >99% of the ORFs were successfully constructed from *h*^-^ (heterothallic) and *h*^90^ (homothallic) parent strains. Importantly, the parent strains have no auxotrophic mutation other than *ura4*^-^, and the uracil auxotrophy conferred by the mutation was converted to uracil prototrophy when the deletion mutants were generated, eliminating the need for nutritional supplements that can serve as a nitrogen source. This feature makes it possible to examine the mutants for growth on various nitrogen sources as described below. Like the previous study on the *S*. *pombe amt* genes [4], a *ura4*^+^ pop-out cassette [14] was used for the gene disruption so that *ura4*^-^ derivatives, which might be useful for molecular genetic analysis, can be constructed by selecting for resistance to 5-fluoroorotic acid.

### Biochemical characterization of *gdh1*Δ, *gdh2*Δ, *gln1*Δ, and *glt1*Δ mutants

In *S*. *pombe*, a putative GS gene was cloned and partially sequenced [19], and mutants defective in NADP-GDH [20], GS [21], or GOGAT [21, 22] activity were isolated and characterized genetically but not molecularly. After the completion of genome sequencing, a genome-wide set of *S*. *pombe* deletion mutants has been constructed [23]; to our knowledge, however, deletion mutants of the genes that are presumed to encode ammonia-metabolizing enzymes had yet to be characterized biochemically.

Enzyme activities of the deletion mutants constructed in this study were determined as described in Materials and methods. Crude extracts prepared from wild-type, *gdh1*Δ, and *gdh2*Δ strains were assayed for NADP-GDH and NAD-GDH activities (Table 2 and S3 Fig). The *gdh1*Δ mutant grown in glucose minimal medium containing ammonium as the nitrogen source completely lacked NADP-GDH activity but showed normal NAD-GDH activity. Conversely, the *gdh2*Δ mutant grown in glutamate completely lacked NAD-GDH activity but showed NADP-GDH activity comparable to that of the wild-type strain. The *gdh1*Δ mutant grown in glutamate and the *gdh2*Δ mutant grown in ammonium exhibited 10%–20% of the NADP-GDH and NAD-GDH activities of the wild-type extracts, respectively; the reason for these activities is not understood. The wild-type strain had higher NADP-GDH activity in ammonium than in glutamate and higher NAD-GDH activity in glutamate than in ammonium, consistent with the biosynthetic role of NADP-GDH and catabolic role of NAD-GDH.

**Table 2:** NADP-GDH and NAD-GDH activities of deletion mutants.

Strain	Genotype	Specific activity (μmol min^-1^ g^-1^)			
		NADP-GDH		NAD-GDH	
		NH_4_^+^	Glu	NH_4_^+^	Glu
HMP126	WT	436.5 ± 11.5	349.2 ± 4.7	107.5 ± 2.9	597.3 ± 12.6
HMP135	*gdh1*Δ	<0	43.7 ± 2.6	70.3 ± 6.3	762.5 ± 22.2
HMP128	*gdh2*Δ	440.6 ± 38.2	261.7 ± 13.1	19.4 ± 9.5	<0

Cells were grown in media containing 10 mM ammonium (NH_4_^+^) or 10 mM glutamate (Glu) as the nitrogen source. Specific activities are expressed as μmol of NADPH (NADP-GDH) or NADH (NAD-GDH) oxidized per min per g of protein. Assays were carried out in triplicate, and mean and standard deviation are shown. The value <0 indicates cases where the blank subtraction resulted in negative values. Similar results were obtained in four (NADP-GDH/NH_4_^+^ and NAD-GDH/NH_4_^+^) or two (NADP-GDH/Glu and NAD-GDH/Glu) experiments.

The results of GS assay are shown in Table 3 and S3 Fig. The *gln1*Δ mutant exhibited approximately 40-fold less activity than the wild type. Since the *gln1*Δ mutant was found to be a glutamine auxotroph (see below), the strain is most likely to be incapable of glutamine production *in vivo*. GOGAT assay revealed that the *glt1*Δ mutant had no GOGAT activity (Table 3 and S3 Fig).

**Table 3:** GS and GOGAT activities of deletion mutants.

Strain	Genotype	Specific activity (μmol min^-1^ g^-1^)			
		GS	GOGAT		
		Gln	NH_4_^+^	Gln	Glu
HMP126	WT	68.2 ± 3.7	50.6 ± 5.8	37.8 ± 8.0	14.6 ± 5.6
HMP141	*gln1*Δ	1.6 ± 0.1	NT	NT	NT
HMP125	*glt1*Δ	NT	<0	<0	<0

Cells were grown in media containing 10 mM ammonium (NH_4_^+^), 10 mM glutamine (Gln), or 10 mM glutamate (Glu) as the nitrogen source. Specific activities are expressed as μmol of γ-glutamyl hydroxamate produced (GS) or NADH oxidized (GOGAT) per min per g of protein. Assays were carried out in triplicate, and mean and standard deviation are shown. The value <0 indicates cases where the blank subtraction resulted in negative values. NT, not tested. Similar results were obtained in four (GS/Gln and GOGAT/NH_4_^+^) or two (GOGAT/Gln and GOGAT/Glu) experiments.

In summary, the enzyme assays demonstrated that the *S*. *pombe gdh1*, *gdh2*, *gln1*, and *glt1* genes encode NADP-GDH, NAD-GDH, GS, and GOGAT, respectively.

### Growth phenotypes of *gdh1*Δ, *gdh2*Δ, *gln1*Δ, and *glt1*Δ mutants

**Fig 2:**
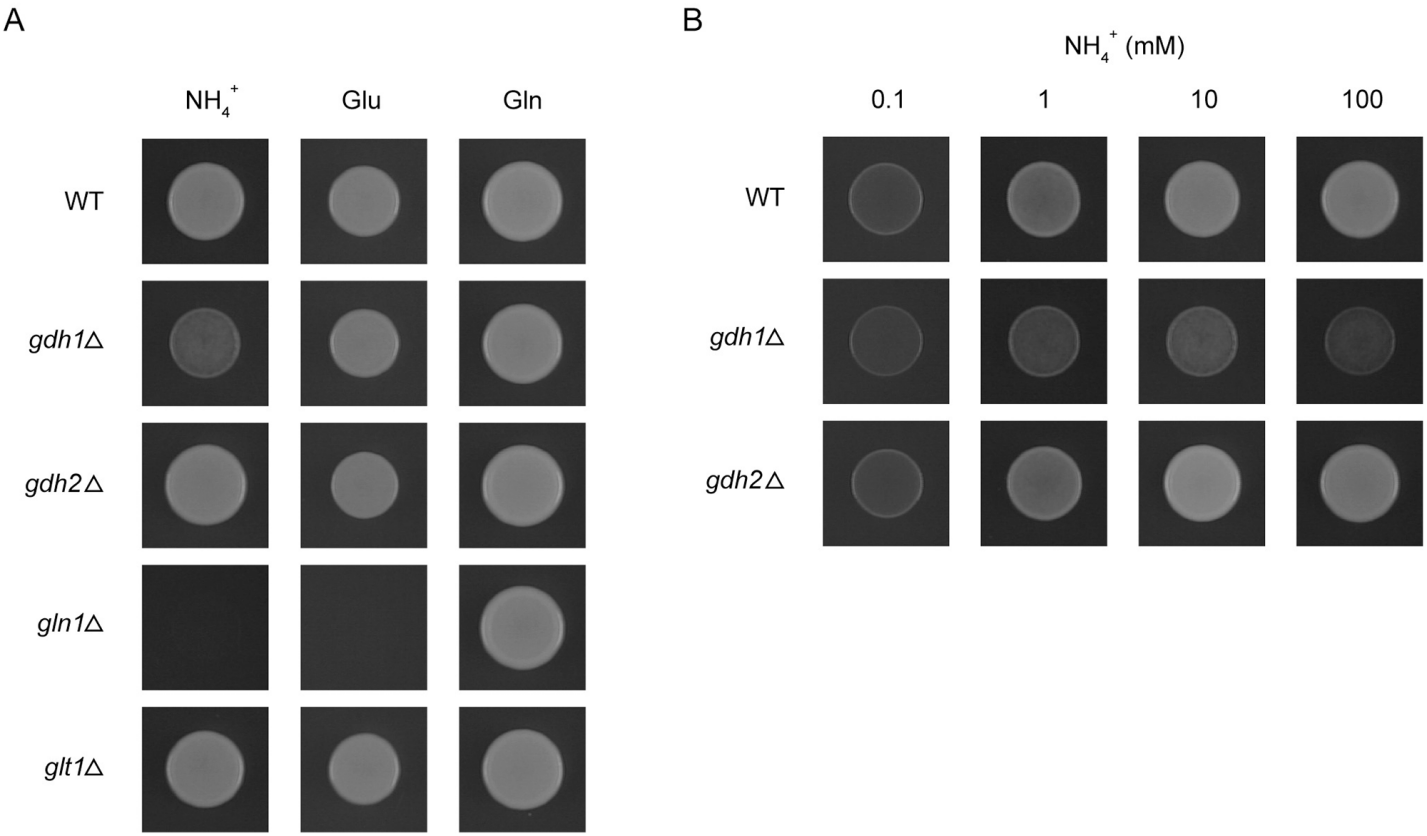
Growth of *gdh1*Δ, *gdh2*Δ, *gln1*Δ, and *glt1*Δ mutants on various nitrogen sources. Five-microliter aliquots of cell suspension (5 x 10^4^ cells) were spotted onto glucose minimal medium containing 10 mM ammonium (NH_4_^+^), 10 mM glutamate (Glu), or 10 mM glutamine (Gln) (A), or 0.1, 1, 10, or 100 mM ammonium (B) as the nitrogen source. The plates were incubated at 30°C for 2 days before photographs were taken. Strains are the same set of strains as used for enzyme assays: HMP126 (WT), HMP135 (*gdh1*Δ), HMP128 (*gdh2*Δ), HMP141 (*gln1*Δ), and HMP125 (*glt1*Δ).

To gain insight into the physiological roles of NADP-GDH, NAD-GDH, GS, and GOGAT in *S*. *pombe*, the *gdh1*Δ, *gdh2*Δ, *gln1*Δ, and *glt1*Δ mutants were tested for growth on glucose minimal media containing ammonium, glutamate, or glutamine as the nitrogen source (Fig 2). The most evident growth defect was observed for the *gln1*Δ mutant. The *gln1*Δ mutant was able to grow on glutamine but not on ammonium or glutamate (Fig 2A). The glutamine auxotrophy conferred by the deletion of the *gln1* gene indicates that GS encoded by *gln1* is the only enzyme that catalyzes the formation of glutamine in *S*. *pombe*.

Fig 2A also shows a growth defect of the *gdh1*Δ mutant on ammonium. Interestingly, the defect was more pronounced at higher ammonium concentrations; while little difference in growth was seen between the wild-type strain and the *gdh1*Δ mutant on 0.1 mM ammonium, *gdh1*Δ grew much more slowly than the wild type on 100 mM ammonium (Fig 2B). These growth phenotypes of the *gdh1*Δ mutant suggest that NADP-GDH of *S*. *pombe* plays a major role in ammonium assimilation when extracellular ammonium concentrations are high. Unlike the *gln1*Δ and *gdh1*Δ mutants, the *gdh2*Δ and *glt1*Δ mutants did not exhibit growth defects on 10 mM ammonium, 10 mM glutamate, or 10 mM glutamine.

### Filamentous invasive growth of *gdh1*Δ, *gdh2*Δ, and *glt1*Δ mutants and the effect of increased concentrations of ammonium

**Fig 3:**
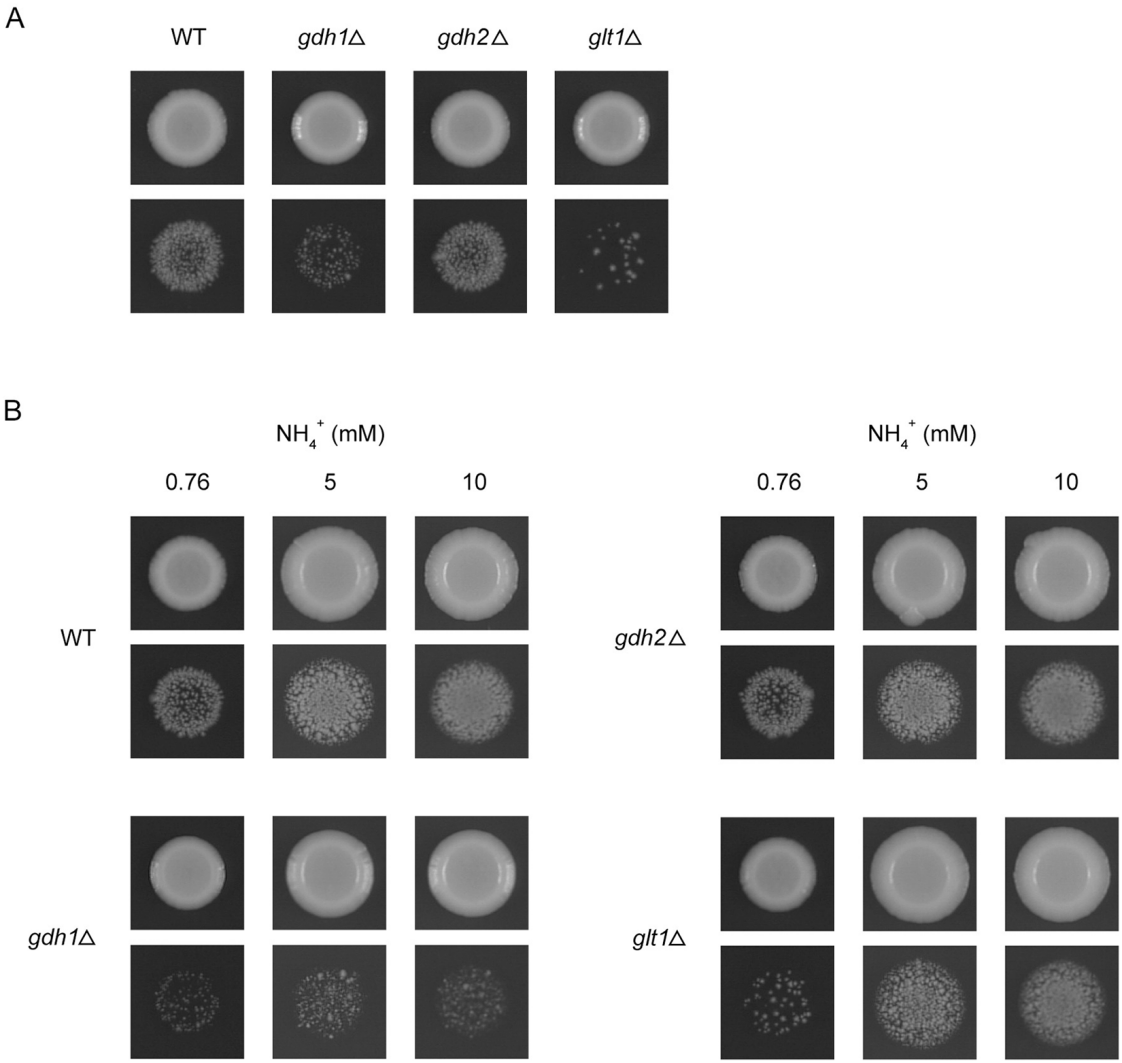
Invasive growth of *gdh1*Δ, *gdh2*Δ, and *glt1*Δ mutants and effect of increasing concentrations of ammonium. Invasive growth was assayed as in Fig 1. (A) HMP126 (WT), HMP135 (*gdh1*Δ), HMP128 (*gdh2*Δ), and HMP125 (*glt1*Δ) were grown on LNB medium. (B) HMP94 (WT), HMP137 (*gdh1*Δ), HMP133 (*gdh2*Δ), and HMP131 (*glt1*Δ) were grown on LNB medium and media in which 0.76 mM NH_4_^+^ in LNB was replaced by 5 or 10 mM NH_4_^+^.

The deletion mutants were then tested for their ability to undergo filamentous invasive growth upon ammonium limitation. The *gln1*Δ mutant was excluded from the test because of its inability to grow on ammonium. As shown in Fig 3A, the wild-type strain exhibited invasive growth after 2 weeks of incubation on LNB agar medium. Whereas the *gdh2*Δ mutant underwent invasive growth comparable to that of the wild-type strain, the *gdh1*Δ and *glt1*Δ mutants showed reduced invasive growth. The *gdh1*Δ mutant also exhibited slower surface growth on LNB, which was not evident after 14 day incubation (Fig 3A).

Next, we tested whether increased concentrations of ammonium in the medium suppressed the invasive growth defect of *gdh1*Δ and *glt1*Δ mutants, as in the case of the *amt1*Δ mutant shown in Fig 1. Interestingly, the *gdh1*Δ and *glt1*Δ mutants behaved differently at higher ammonium concentrations. We observed an enhancement in invasive growth by increasing the ammonium concentration for the *glt1*Δ mutant but not for the *gdh1*Δ mutant (Fig 3B).

## Discussion

*S*. *cerevisiae* has sensing systems to respond to the availability of nutrients in the environment [24, 25]. Some membrane proteins that share homology with transporters but lack transport capacity are known to function as sensors. These include Snf3 and Ssy1 in *S*. *cerevisiae*, which are involved in glucose and amino acid sensing, respectively. Unlike these transporter homologs, certain transporters have been proposed to have a receptor function in addition to their transport function [26]. Among them are the general amino acid permease Gap1 and the high-affinity ammonium transporter Mep2 of *S*. *cerevisiae*. Mep2 is required for diploid pseudohyphal growth in response to ammonium limitation [1].

*S*. *pombe* was found to form microcolonies of filamentously growing cells underneath the surface of a low-ammonium medium ([4] and S4 Fig). The filaments have constrictions at the septal junctions and thus cannot be called true hyphae, which have no such constrictions and have parallel sides along their entire length [27]. In this regard, *S*. *pombe* differs from the related fission yeast *Schizosaccharomyces japonicus*, which shows yeast-to-hypha transition [28]. The growth pattern of the cells in the filamentous structures of *S*. *pombe* is also different from that in the pseudohyphae of *S*. *cerevisiae* [29]. A major difference is the direction of growth of subapical cells. Pseudohyphal cells are unipolar, producing their second and subsequent buds only from the end opposite the junction with their mother [30], that is, the subapical cells of the *S*. *cerevisiae* pseudohyphae grow by budding in the same direction as the apical cell. In contrast, the subapical cells of the *S*. *pombe* filamentous structures usually grow in the direction away from the apical cell [31 and S4 Fig]. Because the filamentous structures of *S*. *pombe* differ from both hyphae and pseudohyphae, we have referred to the *S*. *pombe* invasive growth as filamentous invasive growth. The formation of microcolonies in the agar indicates that agar invasion is a rare event. It appears that only a small number of cells penetrate the agar and grow filamentously, although the underlying mechanism is not understood.

The *S*. *pombe* strains used in our previous study were homothallic *h*^90^ strains [4], which are capable of switching of the mating types between *h*^+^ and *h*^-^, so that mating can occur within a culture. In the present study, we found that heterothallic *h*^-^ strains also underwent invasive growth in response to ammonium limitation (Fig 3A). Therefore, the ammonium limitation-induced filamentous invasive growth in *S*. *pombe*, unlike the pseudohyphal growth in *S*. *cerevisiae*, is a morphological transition that occurs in haploid cells. However, the possibility has not been excluded that diploid *S*. *pombe* cells can also undergo invasive growth as in case of *S*. *japonicus* [32].

We found that the *amt1*Δ mutant became capable of undergoing invasive growth when the ammonium concentration in the medium was increased from 0.76 to 10 mM, revealing that Amt1 is dispensable for filamentous invasive growth at higher ammonium concentrations. The observation suggests that the nitrogen status of the cell, rather than the Amt1 itself, is important for the morphological transition.

In the course of this study, deletion mutants of the *S*. *pombe gdh1*, *gdh2*, *gln1*, and *glt1* genes were generated and were demonstrated to encode NADP-GDH, NAD-GDH, GS, and GOGAT, respectively. Because these strains have no nutritional requirements, they are suitable for analysis of the physiological function of the genes. For molecular genetic analysis, *ura4*^-^ derivatives, which can easily be generated from the strains, might be useful.

The *gdh1*Δ and *glt1*Δ mutants showed reduced invasive growth compared to the wild-type strains. The *gdh1* and *glt1* genes encode NADP-GDH and GOGAT, respectively, and both the enzymes catalyze the production of glutamate, suggesting a possibility that the intracellular level of glutamate may be important in the induction of invasive growth. Increasing the ammonium concentration in the medium suppressed the invasive growth defect of the *glt1*Δ mutant, but not the *gdh1*Δ mutant, indicating that NADP-GDH is necessary for an effect of increasing concentrations of ammonium.

cAMP has shown to regulate the fungal morphological transitions [1, 4, 13, 33, 34]. cAMP acts negatively in a switch from yeast to hyphal growth of *S*. *japonicus* but positively in a switch from yeast to filamentous invasive growth of *S*. *pombe*. In *S*. *pombe*, addition of cAMP to the medium lead to an enhancement of filamentous invasive growth [4, 13]. Thus the ammonia metabolism might somehow be connected to the cAMP pathway. An as yet unidentified signal produced through ammonia metabolism may activate the cAMP pathway. Conversely, the cAMP pathway may stimulate the production of the signal by regulating an ammonia-metabolizing enzyme.

Ammonium limitation is not the only stimulus that induces morphological transition in *S*. *pombe*. It has been reported that glutamine limitation [4] and high iron concentrations [35] can also trigger invasive growth of *S*. *pombe*. Because glutamine is a key metabolite in ammonia assimilation, ammonium limitation-induced invasive growth and glutamine limitation-induced invasive growth may share a signaling pathway.

The present study characterized the *S*. *pombe* genes that encode enzymes involved in ammonia metabolism and suggested their possible role in the ammonium limitation-induced filamentous invasive growth. Our results point to the importance of the nitrogen status of the cell in the induction of the morphological transition. It will be of interest to determine the concentration of metabolites in the deletion mutants constructed in this study. The deletion mutants would also serve as valuable tools for investigating the physiological roles of the ammonia-metabolizing enzymes in *S*. *pombe*.

## Supporting information

S1 Fig Location of the primers for the construction of *gdh1*Δ, *gdh2*Δ, *gln1*Δ, and *glt1*Δ mutants. The arrows above the genes indicate the primer pairs used for amplifying 5-upstream and 3-downstream sequences, and the arrows below the genes indicate the external and internal primer pairs used for verifying the disruption. For example, IF1 on the *gdh1* gene denotes the forward primer gdh1-IF1 whose 5’ terminus is -755 relative to the A of the initiation codon. Primers are not drawn to scale. The brackets show the deleted regions.
(PDF)

S2 Fig Verification of *gdh1*Δ, *gdh2*Δ, *gln1*Δ, and *glt1*Δ mutants. PCR was performed with the external (left panels) and internal (right panels) primer pairs of the *gdh1* (A), *gdh2* (B), *gln1* (C), and *glt1* (D) genes, using genomic DNA as template. The PCR products were analyzed on a 0.8% (left panels) or 1.2% (right panels) agarose gel followed by staining with ethidium bromide. M, DNA size marker. The expected sizes of the PCR products amplified from the wild-type allele with the external primer pairs for the *gdh1*, *gdh2*, *gln1*, and *glt1* genes are 2846, 5487, 2446, and 8166 bp, and those with the internal primer pairs are 886, 1146, 521 and 695 bp, respectively. The external primer pair for *glt1* yielded an additional band just below a band with the expected size (D, left panel). Strains are a set of *h*^-^ strains, HMP126 (WT), HMP135 (*gdh1*Δ), HMP128 (*gdh2*Δ), HMP141 (*gln1*Δ), and HMP125 (*glt1*Δ). Similar results were obtained for a set of *h*^90^ strains, HMP94, HMP137, HMP133, HMP143, and HMP131.
(PDF)

S3 Fig Time courses of absorbance at 340 nm used for the calculation of the enzyme activities. One of each triplicate reaction is shown. (A) NADP-GDH assay of the extracts from wild-type (HMP126) and *gdh1*Δ (HMP135) cells grown in 10 mM ammonium medium. (B) NAD-GDH assay of the extracts from wild-type (HMP126) and *gdh2*Δ (HMP128) cells grown in 10 mM glutamate medium. (C) GOGAT assay of the extracts from wild-type (HMP126) and *glt1*Δ (HMP125) cells grown in 10 mM ammonium medium. Data were obtained using a kinetics program for the Shimadzu UVmini-1240 Spectrophotometer. Assay conditions are described in Materials and methods.
(PDF)

S4 Fig Morphology of *S*. *pombe* cells growing in the agar. HMP94 (WT) was grown on LNB medium, washed off the surface of the agar, and photographed under a microscope. The arrowhead in panel d indicates a cell, one end of which appears to grow in the direction away from the apical cell. The scale bars represent 50 μm (a) and 10 μm (b, c, d).
(PDF)

S1 Table PCR primers used in this study. (PDF)

S2 Table Genes encoding ammonia-metabolizing enzymes in *Schizosaccharomyces pombe* and *Saccharomyces cerevisiae*. (PDF)
