# Supplementary material for: Filamentous invasive growth of mutants of the genes encoding ammonia-metabolizing enzymes in the fission yeast Schizosaccharomyces pombe
Source: PLoS One. 2017 Oct 5;12(10):e0186028. doi: 10.1371/journal.pone.0186028 (PMC5628922; doi:10.1371/journal.pone.0186028)

S1 Fig. Location of the primers for the construction of *gdh1*Δ, *gdh2*Δ, *gln1*Δ, and *glt1*Δ mutants

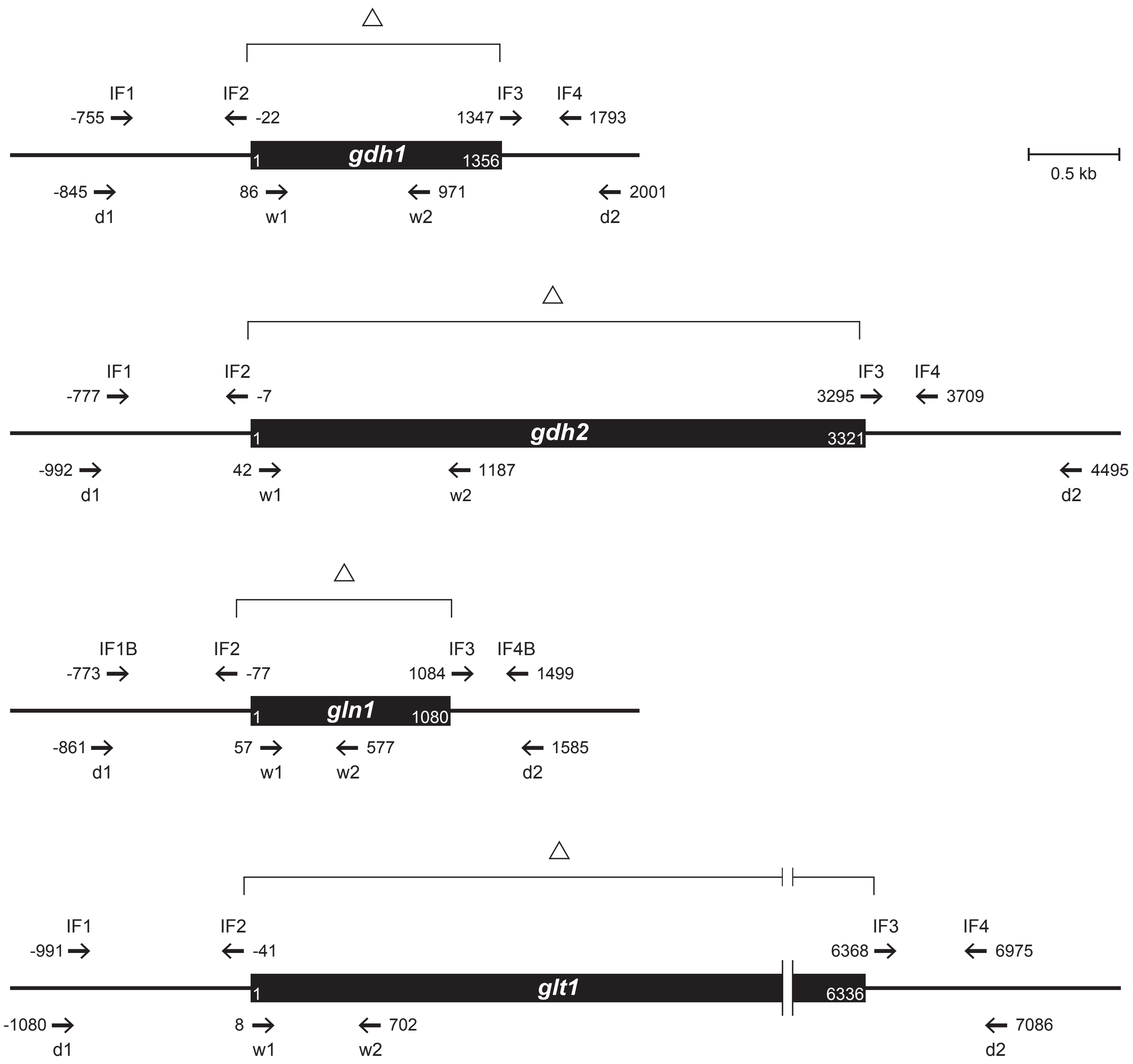

Supplement: S1 Fig — The arrows above the genes indicate the primer pairs used for amplifying 5-upstream and 3-downstream sequences, and the arrows below the genes indicate the external and internal primer pairs used for verifying the disruption. For example, IF1 on the gdh1 gene denotes the forward primer gdh1-IF1 whose 5’ terminus is -755 relative to the A of the initiation codon. Primers are not drawn to scale. The brackets show the deleted regions. (PDF) [file pone.0186028.s001.pdf]
