# Supplementary figures and images for: Filamentous invasive growth of mutants of the genes encoding ammonia-metabolizing enzymes in the fission yeast Schizosaccharomyces pombe
Source: PLoS One. 2017 Oct 5;12(10):e0186028. doi: 10.1371/journal.pone.0186028 (PMC5628922; doi:10.1371/journal.pone.0186028)

**S2 Fig.** Verification of *gdh1Δ*, *gdh2Δ*, *gln1Δ*, and *glt1Δ* mutants

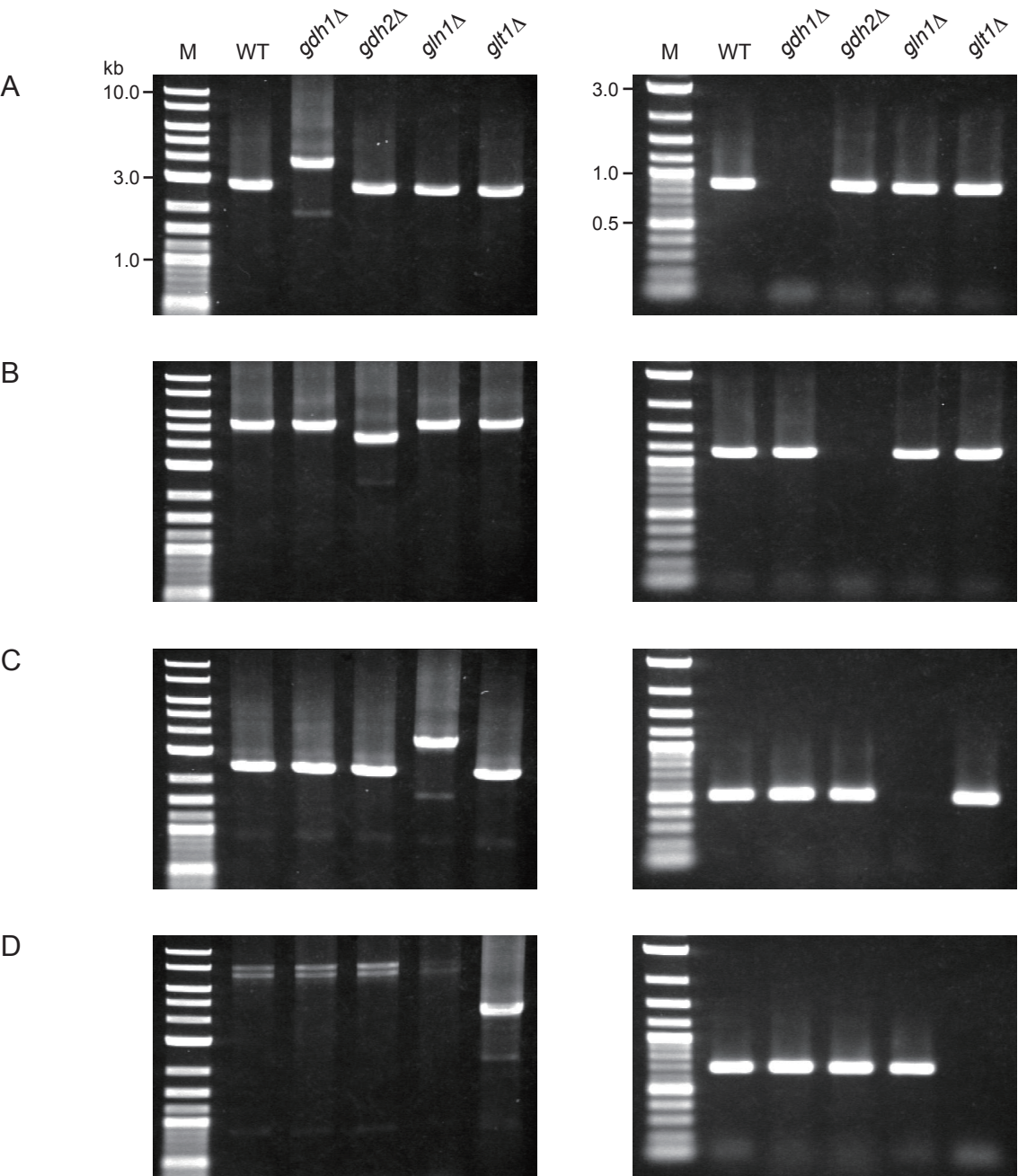

Supplement: S2 Fig — PCR was performed with the external (left panels) and internal (right panels) primer pairs of the gdh1 (A), gdh2 (B), gln1 (C), and glt1 (D) genes, using genomic DNA as template. The PCR products were analyzed on a 0.8% (left panels) or 1.2% (right panels) agarose gel followed by staining with ethidium bromide. M, DNA size marker. The expected sizes of the PCR products amplified from the wild-type allele with the external primer pairs for the gdh1, gdh2, gln1, and glt1 genes are 2846, 5487, 2446, and 8166 bp, and those with the internal primer pairs are 886, 1146, 521 and 695 bp, respectively. The external primer pair for glt1 yielded an additional band just below a band with the expected size (D, left panel). Strains are a set of h- strains, HMP126 (WT), HMP135 (gdh1Δ), HMP128 (gdh2Δ), HMP141 (gln1Δ), and HMP125 (glt1Δ). Similar results were obtained for a set of h90 strains, HMP94, HMP137, HMP133, HMP143, and HMP131. (PDF) [file pone.0186028.s002.pdf]

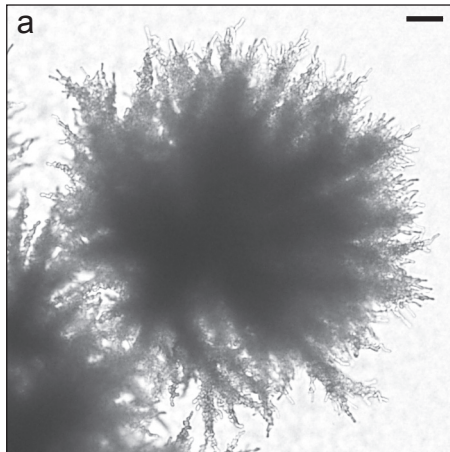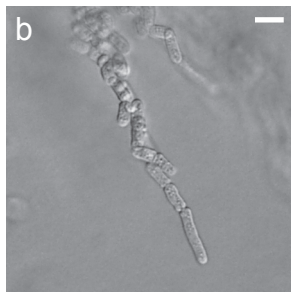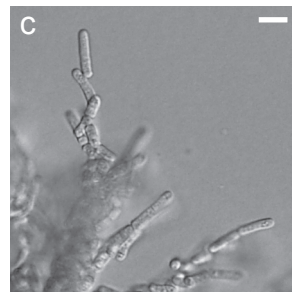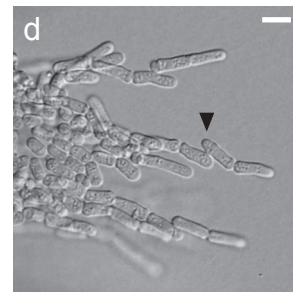

Supplement: S4 Fig — HMP94 (WT) was grown on LNB medium, washed off the surface of the agar, and photographed under a microscope. The arrowhead in panel d indicates a cell, one end of which appears to grow in the direction away from the apical cell. The scale bars represent 50 μm (a) and 10 μm (b, c, d). (PDF) [file pone.0186028.s004.pdf]
