# Supplementary material for: Filamentous invasive growth of mutants of the genes encoding ammonia-metabolizing enzymes in the fission yeast Schizosaccharomyces pombe
Source: PLoS One. 2017 Oct 5;12(10):e0186028. doi: 10.1371/journal.pone.0186028 (PMC5628922; doi:10.1371/journal.pone.0186028)

S3 Fig. Time courses of absorbance at 340 nm used for the calculation of the enzyme activities

A

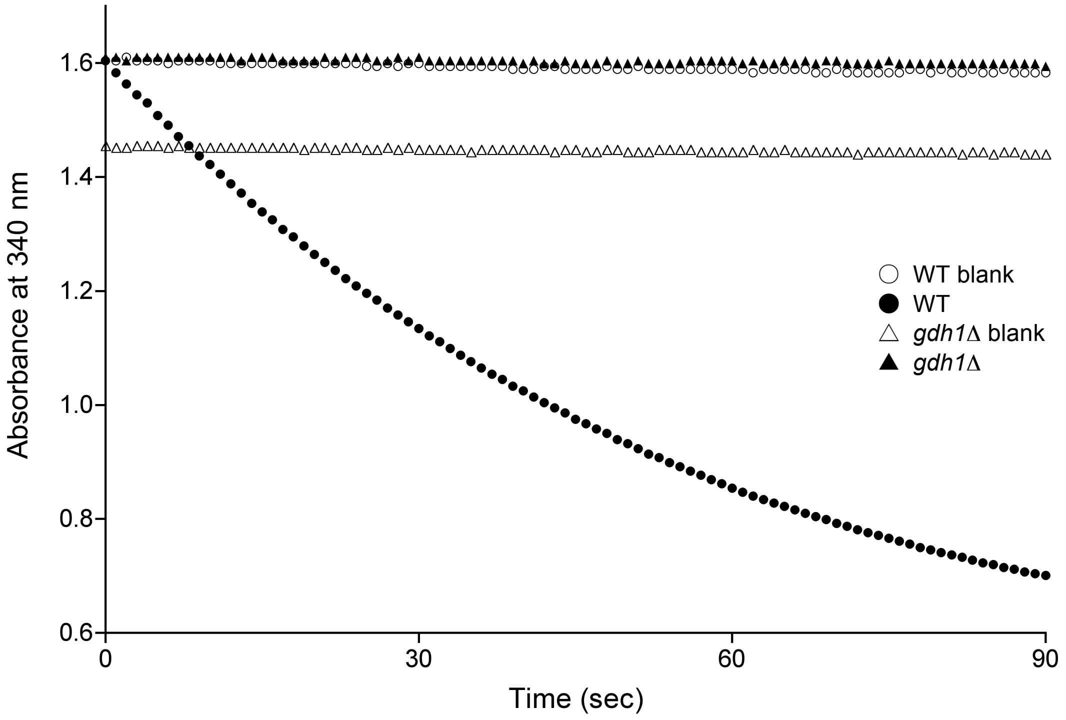

B

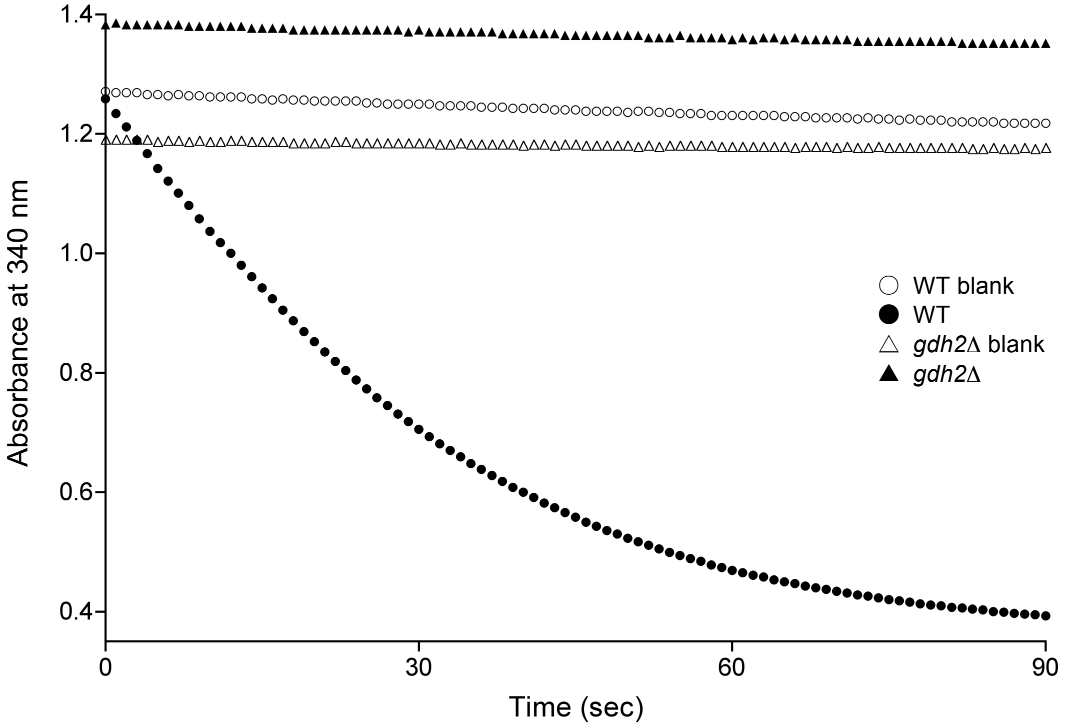

S3 Fig. (continued)

C

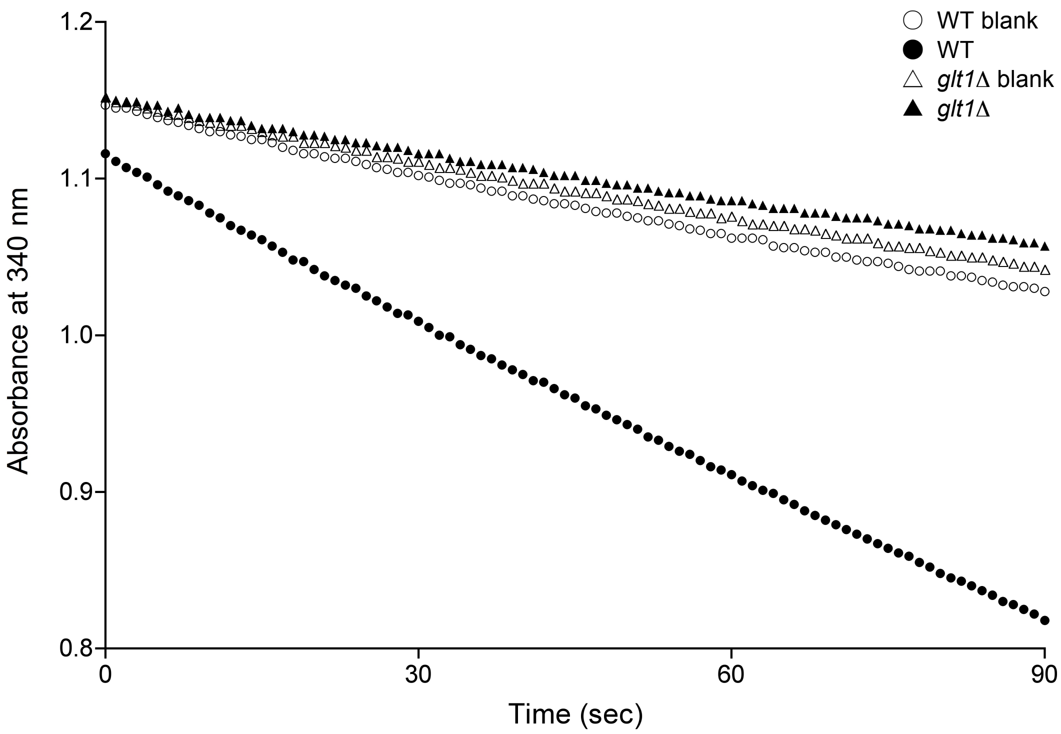

Supplement: S3 Fig — One of each triplicate reaction is shown. (A) NADP-GDH assay of the extracts from wild-type (HMP126) and gdh1Δ (HMP135) cells grown in 10 mM ammonium medium. (B) NAD-GDH assay of the extracts from wild-type (HMP126) and gdh2Δ (HMP128) cells grown in 10 mM glutamate medium. (C) GOGAT assay of the extracts from wild-type (HMP126) and glt1Δ (HMP125) cells grown in 10 mM ammonium medium. Data were obtained using a kinetics program for the Shimadzu UVmini-1240 Spectrophotometer. Assay conditions are described in Materials and methods. (PDF) [file pone.0186028.s003.pdf]
