# Supplementary material for: Filamentous invasive growth of mutants of the genes encoding ammonia-metabolizing enzymes in the fission yeast Schizosaccharomyces pombe
Source: PLoS One. 2017 Oct 5;12(10):e0186028. doi: 10.1371/journal.pone.0186028 (PMC5628922; doi:10.1371/journal.pone.0186028)
Supplement: S1 Table — (PDF) [file pone.0186028.s005.pdf]

S1 Table PCR primers used in this study

Designation	Sequence
Primers for conversion of *ura4-D18* to *ura4*$^{+}$	
ura4-g1	5’- TCTCAAAGTCCTCCTAGTCAATGG-3’
ura4-g2	5’-TCATACAAACGTCTTCAGCTATAC-3’
Primers for In-Fusion cloning for construction of disruption plasmids	
pUC19-1B	5’-AACGTCGTGACTGGGAAAACC-3’
pUC19-2B	5’-CCGCTCACAATTCCACACAAC-3’
ura4F-1	5’-GTAAAACGACGGCCAGTGAATTGTAATACG-3’
ura4F-2	5’-GACCATGATTACGCCAAGCTATTTAGGTG-3’
gdh1-IF1	5’-TGGAATTGTGAGCGGAAGCACCCCATCCCACTAAG-3’
gdh1-IF2	5’-TGGCCGTCGTTTTACAAATCGAAGGTAGTGATATAGAGTG-3’
gdh1-IF3	5’-GGCGTAATCATGGTCCTGGTGGTAGACTTCATTCGTG-3’
gdh1-IF4	5’-CCCAGTCACGACGTTGTCTATTCTTCACATTTTTAACAGC-3’
gdh2-IF1	5’-TGGAATTGTGAGCGGTGAAAGCTGCATCCTACTATGG-3’
gdh2-IF2	5’-TGGCCGTCGTTTTACCAATCTGAATCTTGTCTCTTTGAC-3’
gdh2-IF3	5’-GGCGTAATCATGGTCGAAATGAAAATGCTGAAGGACG-3’
gdh2-IF4	5’-CCCAGTCACGACGTTATTGTAAATGCCACTTGCGTTG-3’
gln1-IF1B	5’-TGGAATTGTGAGCGGTCCCTGGCACTATCTTTAGGC-3’
gln1-IF2	5’-TGGCCGTCGTTTTACAAAACTAGCAACAGAAGAAAACCC-3’
gln1-IF3	5’-GGCGTAATCATGGTCATTTTCCCTCATAATTTAACGTGC-3’
gln1-IF4B	5’-CCCAGTCACGACGTTGTTGTTGACAAACTGATTCGTAAAG-3’
glt1-IF1	5’-TGGAATTGTGAGCGGGCATTGTAGCGGCTTTTGCAC-3’
glt1-IF2	5’-TGGCCGTCGTTTTACTAGTTGGTGTGTTTGAAAAATCG-3’
glt1-IF3	5’-GGCGTAATCATGGTCTTAATGTCTCTGAATATACGGAGC-3’
glt1-IF4	5’-CCCAGTCACGACGTTCTTGTATTTGATCCTGGTAATGC-3’
Primers for preparation of disruption fragments for yeast transformation	
gdh1-7	5’-AAGCACCCCATCCCACTAAG-3’
gdh1-8	5’-GTCTATTCTTCACATTTTTAACAGC-3’
gdh2-7	5’-TGAAAGCTGCATCCTACTATGG-3’
gdh2-8	5’-ATTGTAAATGCCACTTGCGTTG-3’
gln1-7	5’-TCCCTGGCACTATCTTTAGGC-3’
gln1-8	5’-GTTGTTGACAAACTGATTCGTAAAG-3’
glt1-7	5’-GCATTGTAGCGGCTTTTGCAC-3’
glt1-8	5’-CTTGTATTTGATCCTGGTAATGC-3’
External primers for verification of disruption	
gdh1-d1	5’-CCGAAAATCTCCGTAATTGCAG-3’
gdh1-d2	5’-TTGCATGTCTAGAATAAGCTATCG-3’
gdh2-d1	5’-AATCTACCCCTAAAGGAGTTGC-3’
gdh2-d2	5’-GGATTTGACTGATGCTTACGAC-3’
gln1-d1	5’-AATCAACAGAAAGCGAAAATCTTC-3’
gln1-d2	5’-ATTCATTTATTCCATTACGCTGC-3’
glt1-d1	5’-TGCGTTTCTCTGTTTACTTTTGC-3’
glt1-d2	5’-GTTGTCTTTTTCCCTTATGAACC-3’

S1 Table (continued)

Internal primers for verification of disruption	
gdh1-w1	5'-ATCCCGAGTTGAAGCGTGTG-3'
gdh1-w2	5'-GCTTCCTCACCGCTAACTTC-3'
gdh2-w1	5'-TACGACCCAGTTATTGACGG-3'
gdh2-w2	5'-GGCAAGCAGTAAAGTAAAGAGG-3'
gln1-w1	5'-TGCCGATCTTCCTCAAAATGG-3'
gln1-w2	5'-CGGAAATGTTGATACCAGCG-3'
glt1-w1	5'-TCTTATCTTCAGTCCAACCCATC-3'
glt1-w2	5'-ACCGATCAAATGAGAAGACTGC-3'

The primers ura4-g1 and ura4-g2 [4] were used for amplifying a 2.9-kb *ura4*$^+$ fragment from the genomic DNA of FY7507. The primers pUC19-1B and pUC19-2B were used for amplifying linearized pUC19 vector. The primers ura4F-1 and ura4F-2 were used for amplifying the 2.5-kb *ura4*$^+$ cassette from pGEM3ZpBRura4+pBR. The primers gdh1-IF1, gdh1-IF2, gdh2-IF1, gdh2-IF2, gln1-IF1B, gln1-IF2, glt1-IF1 and glt1-IF2 were used for amplifying 5'-upstream sequences from the genomic DNA of FY7406, and gdh1-IF3, gdh1-IF4, gdh2-IF3, gdh2-IF4, gln1-IF3, gln1-IF4B, glt1-IF3 and glt1-IF4 were used for amplifying 3'-downstream sequences from the genomic DNA of FY7406. The underlined 15-bp sequence in gdh1-IF1, gdh2-IF1, gln1-IF1B, and glt1-IF1 is complementary to the 5' end region of pUC19-2B. The underlined 15-bp sequence in gdh1-IF2, gdh2-IF2, gln1-IF2, and glt1-IF2 is complementary to the 5' end region of ura4F-1. The underlined 15-bp sequence in gdh1-IF3, gdh2-IF3, gln1-IF3, and glt1-IF3 is complementary to the 5' end region of ura4F-2. The underlined 15-bp sequence in gdh1-IF4, gdh2-IF4, gln1-IF4B, and glt1-IF4 is complementary to the 5' end region of pUC19-1B.
