# Supplementary material for: Filamentous invasive growth of mutants of the genes encoding ammonia-metabolizing enzymes in the fission yeast Schizosaccharomyces pombe
Source: PLoS One. 2017 Oct 5;12(10):e0186028. doi: 10.1371/journal.pone.0186028 (PMC5628922; doi:10.1371/journal.pone.0186028)
Supplement: S2 Table — (PDF) [file pone.0186028.s006.pdf]

S2 Table Genes encoding ammonia-metabolizing enzymes in *Schizosaccharomyces pombe* and *Saccharomyces cerevisiae*

Enzyme	Gene (product size in amino acids)		Amino acid identity
	S. pombe	*S. cerevisiae*	
NADP-GDH	*gdh1* (451 aa)	*GDH1* (454 aa)	60%
		GDH3 (457 aa)	59%
NAD-GDH	*gdh2* (1106 aa)	*GDH2* (1092 aa)	33%
GS	*gln1* (359 aa)	*GLN1* (370 aa)	65%
GOGAT	*glt1* (2111 aa)	*GLT1* (2145 aa)	58%

The percentage amino acid identity was calculated with the ClustalW program. Systematic gene names: *gdh1*, SPCC622.12c; *gdh2*, SPCC132.04c; *gln1*, SPAC23H4.06; *glt1*, SPAPB1E7.07; *GDH1*, YOR375C; *GDH2*, YDL215C; *GDH3*, YAL062W; *GLN1*, YPR035W; *GLT1*, YDL171C.
